# Harnessing the Versatility of Bacterial Collagen to Improve the Chondrogenic Potential of Porous Collagen Scaffolds

**DOI:** 10.1002/adhm.201600136

**Published:** 2016-05-24

**Authors:** Paresh A. Parmar, Jean-Philippe St-Pierre, Lesley W. Chow, Jennifer L. Puetzer, Violet Stoichevska, Yong Y. Peng, Jerome A. Werkmeister, John A. M. Ramshaw, Molly M. Stevens

**Affiliations:** Department of Materials, Department of Bioengineering, Institute of Biomedical Engineering, Imperial College London SW7 2AZ, UK; The Commonwealth Scientific and Industrial Research Organisation (CSIRO) Manufacturing, Bayview Avenue, Clayton, Victoria 3169, Australia; Department of Bioengineering Institute of Biomedical Engineering Imperial College London, SW7 2AZ, UK; Department of Bioengineering Institute of Biomedical Engineering Imperial College London, SW7 2AZ, UK; Department of Bioengineering Institute of Biomedical Engineering Imperial College London, SW7 2AZ, UK; The Commonwealth Scientific and Industrial Research Organisation (CSIRO) Manufacturing, Bayview Avenue, Clayton, Victoria 3169, Australia; The Commonwealth Scientific and Industrial Research Organisation (CSIRO) Manufacturing, Bayview Avenue, Clayton, Victoria 3169, Australia; The Commonwealth Scientific and Industrial Research Organisation (CSIRO) Manufacturing, Bayview Avenue, Clayton, Victoria 3169, Australia; The Commonwealth Scientific and Industrial Research Organisation (CSIRO) Manufacturing, Bayview Avenue, Clayton, Victoria 3169, Australia; Department of Bioengineering Institute of Biomedical Engineering Imperial College London, SW7 2AZ, UK

## Abstract

Collagen I foams are used in the clinic as scaffolds to promote articular cartilage repair as they provide a bioactive environment for cells with chondrogenic potential. However, collagen I as a base material does not allow for precise control over bioactivity. Alternatively, recombinant bacterial collagens can be used as “blank slate” collagen molecules to offer a versatile platform for incorporation of selected bioactive sequences and fabrication into 3D scaffolds. Here, we show the potential of Streptococcal collagen-like 2 (Scl2) protein foams modified with peptides designed to specifically and noncovalently bind hyaluronic acid and chondroitin sulfate to improve chondrogenesis of human mesenchymal stem cells (hMSCs) compared to collagen I foams. Specific compositions of functionalized Scl2 foams lead to improved chondrogenesis compared to both nonfunctionalized Scl2 and collagen I foams, as indicated by gene expression, extracellular matrix accumulation, and compression moduli. hMSCs cultured in functionalized Scl2 foams exhibit decreased collagens I and X gene and protein expression, suggesting an advantage over collagen I foams in promoting a chondrocytic phenotype. These highly modular foams can be further modified to improve specific aspects chondrogenesis. As such, these scaffolds also have the potential to be tailored for other regenerative medicine applications.

## 1 Introduction

Articular cartilage is a highly complex and dynamic connective tissue that covers the surfaces of bones in synovial joints,[1] where it provides a low-friction, wear-resistant surface and contributes to load transmission to the underlying subchondral bone.[2] The unique biochemical microenvironment of articular cartilage is intimately linked with the biological function of the tissue and cell-regulated homeostasis.[3] While articular cartilage can perform these self-regulating functions throughout the life of a healthy individual, trauma and/or disease leads to alterations in this environment and causes progressive degeneration of the tissue.[1–3] This process is made worse by articular cartilage’s limited capacity for self-repair and regeneration, which is partly due to its avascular and aneural nature and the inability of resident cells to migrate to the site of injury.[4,5]

A number of treatments have been developed to manage the symptoms of cartilage damage and stimulate articular cartilage repair and/or regeneration. These can be divided into two main categories: (1) nonsurgical approaches such as nonsteroidal anti-inflammatory drugs (NSAIDs),[6] weight loss/joint strengthening, as well as visco-supplementation,[7] and (2) surgical interventions including microfracture,[8] autologous chondrocyte implantation (ACI),[5] mosaicplasty,[1,3] and periosteal transplantation.[9] Cell-based therapies often deliver short-term recovery in joint mobility and pain relief to patients, but the long-term benefits remain elusive.[10] This is often attributed to the quality of the repair tissue, which does not exhibit the same biomechanical composition, organization, and associated mechanical behavior as that of the native tissue, eventually leading to degeneration and failure of the repair tissue. In recent years, significant efforts have been made to improve the outcome of these cell-based strategies through tissue engineering principles.[11–15] Notably, a number of scaffolds have been developed to improve the outcome of ACI and microfracture approaches. These techniques are referred to as matrix-induced autologous chondrocyte implantation (MACI).[16–21] Currently, there are several different types of scaffolds used for MACI in clinical settings that are based on hyaluronic acid (HA) (Hyalograft C),[22] collagen-chondroitin sulfate (CS) (Novocart),[23] fibrin combined with polyglycolic/polylactic acid and polydioxanone (Bioseed C),[24] agarose–alginate (Cartipatch),[25] or collagen type I.[17] Collagen-based scaffolds are most commonly employed for MACI because they have been shown to stimulate the synthesis of glycosaminoglycans (GAGs) and collagen type II,[26,27] the two main extracellular matrix (ECM) components in articular cartilage.[28,29] Collagen type I foams in particular have been used extensively because of their biocompatibility and hydrophilic nature.[30–36] Furthermore, collagen type I foams have inherent bioactivity and cell biorecognition features, which are reported as advantageous. However, collagen type I is not the major collagenous component of articular cartilage matrix and as such may contain bioactive sites that do not facilitate chondrogenesis. A number of studies have also demonstrated a loss of chondrogenic phenotype in resident cells within collagen type I-based scaffolds over a prolonged culture period, leading to the eventual upregulation of hypertrophic markers.[22,34,37]

Recently, recombinantly synthesized bacterial proteins, such as Streptococcus collagen-like 2 (Scl2) protein found on the cell surface of *Streptoccocus pyogenes*,[38] have been investigated for their potential use in tissue engineering applications.[34,39] Similar to mammalian collagens, Scl2 proteins are characterized by a triple helical conformation consisting of the characteristic repeating (Gly–Xaa–Yaa)_n_ units.[34,40,41] Interestingly, Scl2 proteins do not contain cell-binding or bioactive sequences within their backbone and thus effectively offer a biologically “blank collagen template” onto which modifications can be made to systematically integrate carefully selected biological sequences to direct specific cell behavior.[42] Scl2 proteins offer an alternative to mammalian type I collagen because they do not require post-translational modification and exhibit minimal batch-to-batch variation in quality, predictability of performance, and purity. These proteins have also been shown to be noncytotoxic and nonimmunogenic.[41,42] Thus, Scl2 proteins offer the opportunity to tailor functionality to manipulate specific cellular responses. The incorporation of bioactive moieties can be achieved either by tethering from the backbone[43] or integrating the peptide sequence within the collagen backbone via site-directed mutagenesis during Scl2 production,[42] making Scl2 proteins an advantageous alternative to collagen type I for scaffold fabrication.

In this work, we developed porous foams based on these recombinant Scl2 proteins with peptide sequences incorporated in the backbone that specifically bind HA or CS. GAGs such as HA and CS are abundantly present in the native cartilage ECM and are known to play significant roles in a variety of cell–ECM, cell–cell, and protein interactions.[44,45] Their presence in native articular cartilage is particularly important for tissue mechanics and biological function. Our group has recently shown that the HA-binding and CS-binding peptides can specifically and dynamically bind HA and CS within a single scaffold, mimicking the dynamic nature of the ECM.[44,45] In this work, we demonstrate the potential of Scl2 protein foams containing varying ratios of GAG-binding peptides to modulate an improved chondrogenic response by human mesenchymal stem cells (hMSCs) compared to collagen type I foams. These highly versatile foams can be further modified through the incorporation of additional bioactivity to the Scl2 backbone to finely tune the cellular response and recapitulate the complexity of the native microenvironment. As such, these scaffolds have the potential to also be adapted for other applications in regenerative medicine and tissue engineering.

## 2 Results and Discussion

### 2.1 Characterization of Collagen Foams

#### 2.1.1 Morphological Characterization

**Figure 1:**
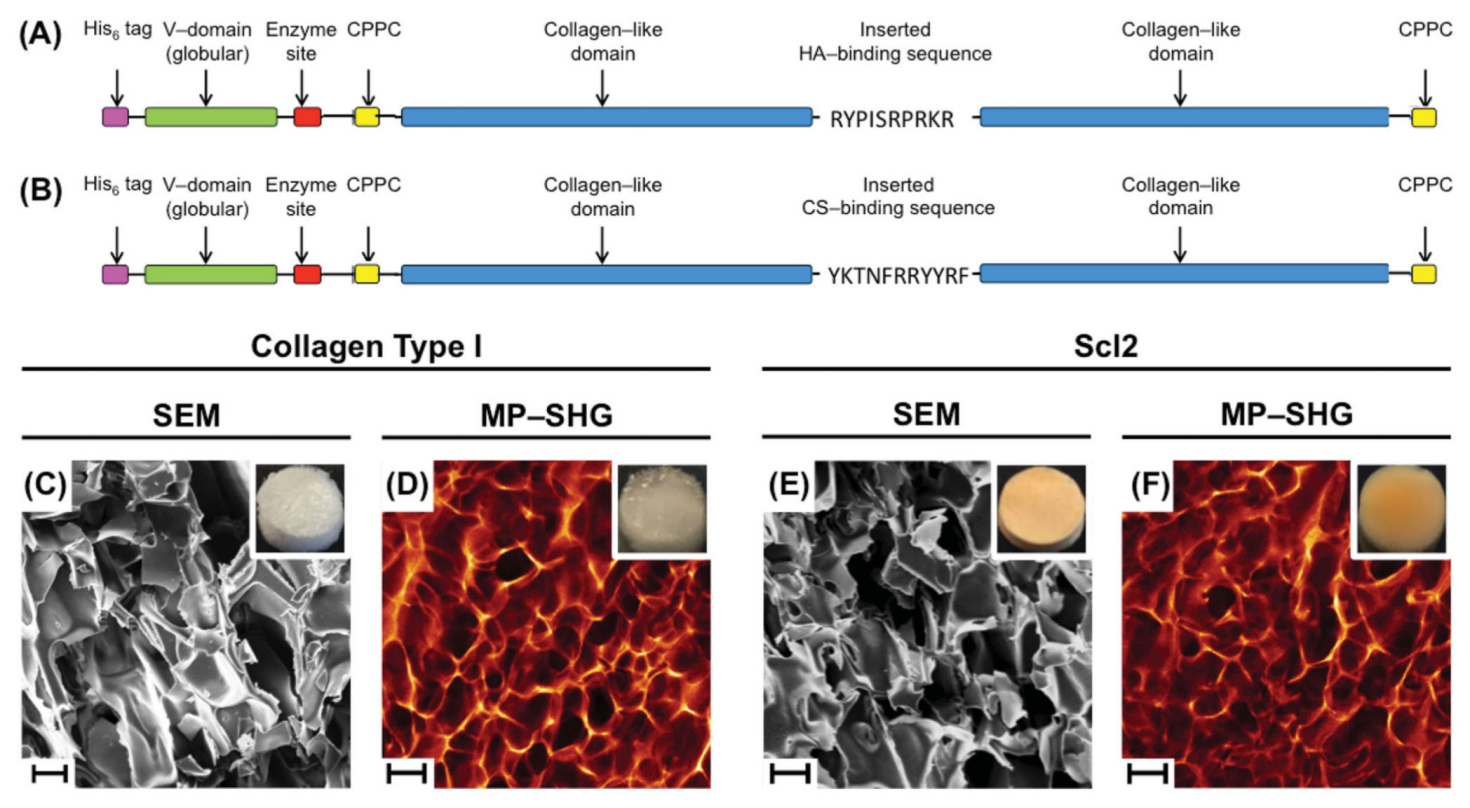
Schematic diagrams of Streptococcal collagen-like 2 (Scl2) protein constructs containing A) HA-binding and B) CS-binding peptide sequences. The “CPPC” domains represent the amino acid sequences inserted at the N and C termini to enhance stability of each construct. Collagen foams morphology. Representative SEM images of C) collagen type I and E) Scl2 foams (scale bars are 100 μm). Representative MP–SHG images of D) collagen type I and F) Scl2 foams (scale bars are 200 μm). Insets show photographs of collagen foams of 8 mm in diameter.

Collagen foams were prepared with blank Scl2, HA-Scl2, CS-Scl2, mixtures of HA-Scl2 and CS-Scl2, and collagen type I, and then imaged using scanning electron microscopy (SEM) and multiphoton second harmonic generation (MP-SHG) (Figure 1, Figure S1, and Figure S2, Supporting Information). No differences in morphology were observed between the different types of foams including those made with collagen type I versus Scl2. Additionally, FTIR spectroscopy (Figure S3, Supporting Information) confirmed no identifiable differences between all collagen foams. A characteristic IR transmittance peak at 1630 cm^−1^ (amide C==O) was present in all samples. All foams were subjected to a dehydrothermal crosslinking treatment, whereby the resultant intermolecular crosslinks formed via condensation reactions likely locked in the collagen molecules resulting in the observed morphologies.[46–48] Multiple studies have shown that the majority of the collagen molecules maintain their triple helical conformation under the crosslinking conditions used in the present study. Previous studies have also demonstrated the presence of a triple helix in Scl2 molecules that have been modified to incorporate integrin-binding or heparin-binding domains in the backbone.[39,42]

#### 2.1.2 Mechanical Characterization

For all foams, the collagen concentration and crosslinking treatment were kept constant to produce structures with similar mechanical properties to facilitate appropriate comparison of cellular response on the different foam compositions.[49–51] The compression moduli of the foams were determined using unconfined DMA and found to range between ≈2.9–3.6 kPa at 1 Hz (Figure S4, Supporting Information). There were no statistical differences between compression moduli of the different bacterial collagen and collagen type I foam formulations. The foams also exhibited increased compression moduli with increasing frequency from 0.1 Hz (≈1.1–1.8 kPa) to 10 Hz (≈5.5–6.2 kPa).

#### 2.1.3 GAG-Binding and Swelling Behavior

**Figure 2:**
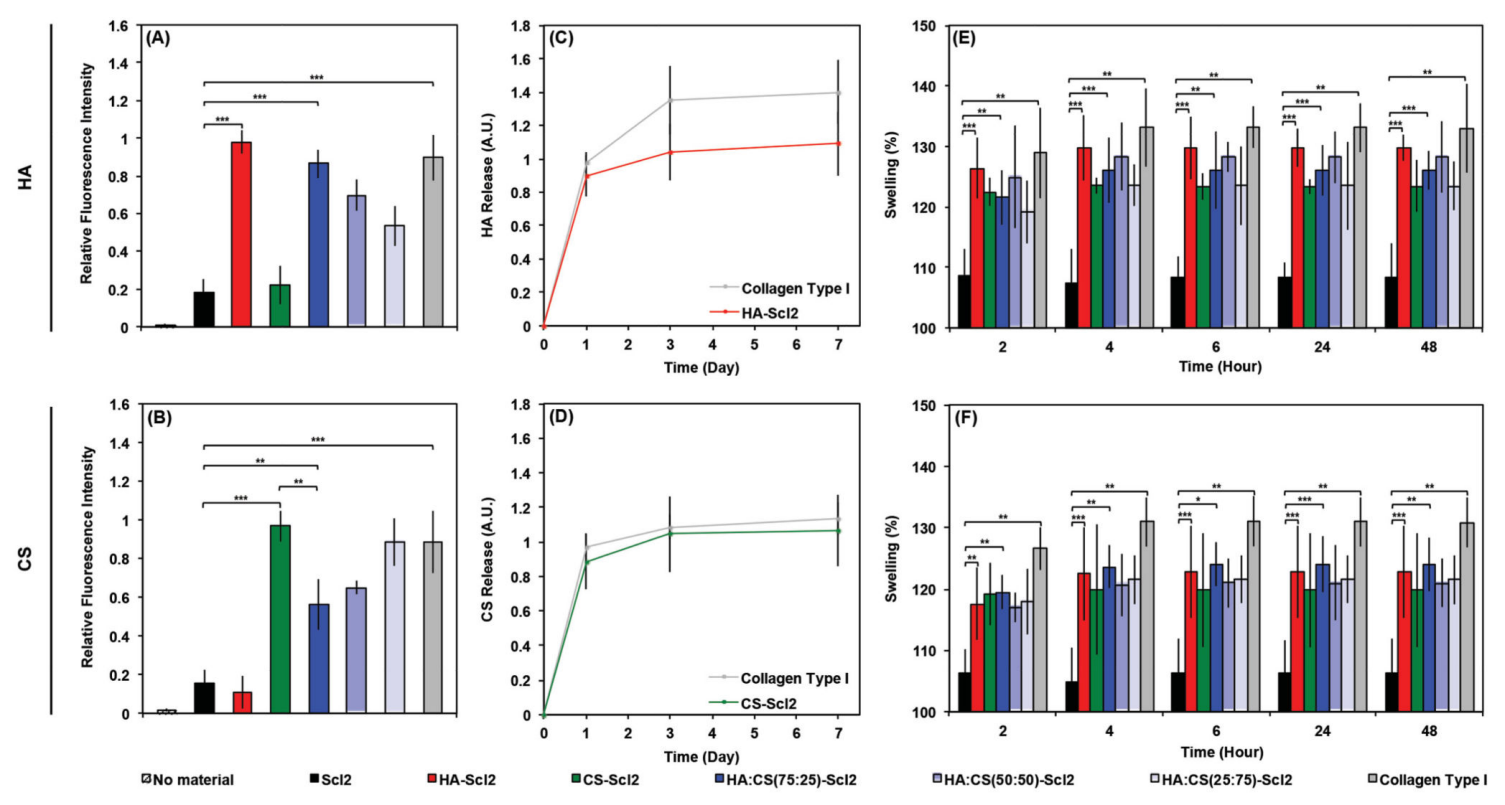
GAG binding on collagen foams. Binding of fluorescently labeled A) HA and B) CS to bacterial collagen and collagen type I foams. Empty wells were used as negative control denoted “no material.” Release of fluorescently labeled C) HA and D) CS from collagen foams over 1 week. Swelling behavior of collagen foams after preincubation with E) HA and F) CS expressed as a percentage of the swelling of collagen foams without HA and CS. Values represent means ± SD. *p < 0.05, **p < 0.01, ***p < 0.001 (*n* = 3).

Bacterial collagen proteins containing HA-binding and CS-binding sequences demonstrated specific binding to HA and CS, respectively (Figure 2A,B), compared to the blank Scl2 control and proteins functionalized with a different binding moiety. Interestingly, no significant differences were measured in HA and CS binding between the collagen type I proteins and the HA-Scl2 and CS-Scl2 proteins, respectively. Collagen type I is not known to contain reported HA- or CS-specific binding sequences, which suggests that the significant amount of HA and CS binding was due to nonspecific binding, most likely through interactions with other interactive moieties present on collagen type I rather than charge effects caused by the collagen type I and Scl2 molecules.[30–32] At a neutral pH, the amino acids present on collagen type I molecules are known to have a net charge close to the isoelectric point.[52,53] For this reason, it is unlikely that HA and CS, highly negatively charged molecules, would bind to the collagen type I molecules through charge effects alone. A small amount of nonspecific GAG binding to the bacterial collagen proteins was also observed on the HA-Scl2 and CS-Scl2 proteins; however, this binding was not significant compared to the blank Scl2 protein. Importantly, specific GAG binding could be modulated by controlling the relative ratio of the HA-binding to CS-binding peptides, which allows an element of control to the design of bacterial collagen foams that is not possible with collagen type I foams. Moreover, the HA-Scl2 and CS-Scl2 foams exhibited comparable binding and release of HA and CS with the collagen type I foams (Figure 2C,D), respectively. The binding and release of GAGs are likely to be a dynamic process during culture.

Collagen type I and bacterial collagen foams containing HA-binding and/or CS-binding peptides swelled significantly more than the blank Scl2 foams following a 48 h incubation with HA or CS compared to incubation in PBS without exogenous GAGs (Figure 2E,F). These results are in agreement with the GAG-binding findings (Figure 2A,D) as GAGs are known to draw in significant amounts of water.[43,54,55] Interestingly, in the absence of HA or CS, all bacterial collagen foams swelled significantly more than the collagen type I foam at 48 h with the exception of the blank Scl2 foams (Figure S5, Supporting Information). As the swelling behavior of the each foam represents its increase in size, it is likely a reflection of a change at the structural level. As expected, MP–SHG images of all functionalized foams and collagen type I foams demonstrated an expected increase in pore size following incubation with HA or CS compared to the blank Scl2 foams (Figure S2H–U, Supporting Information).

#### 2.1.4 Degradation Kinetics

**Figure 3:**
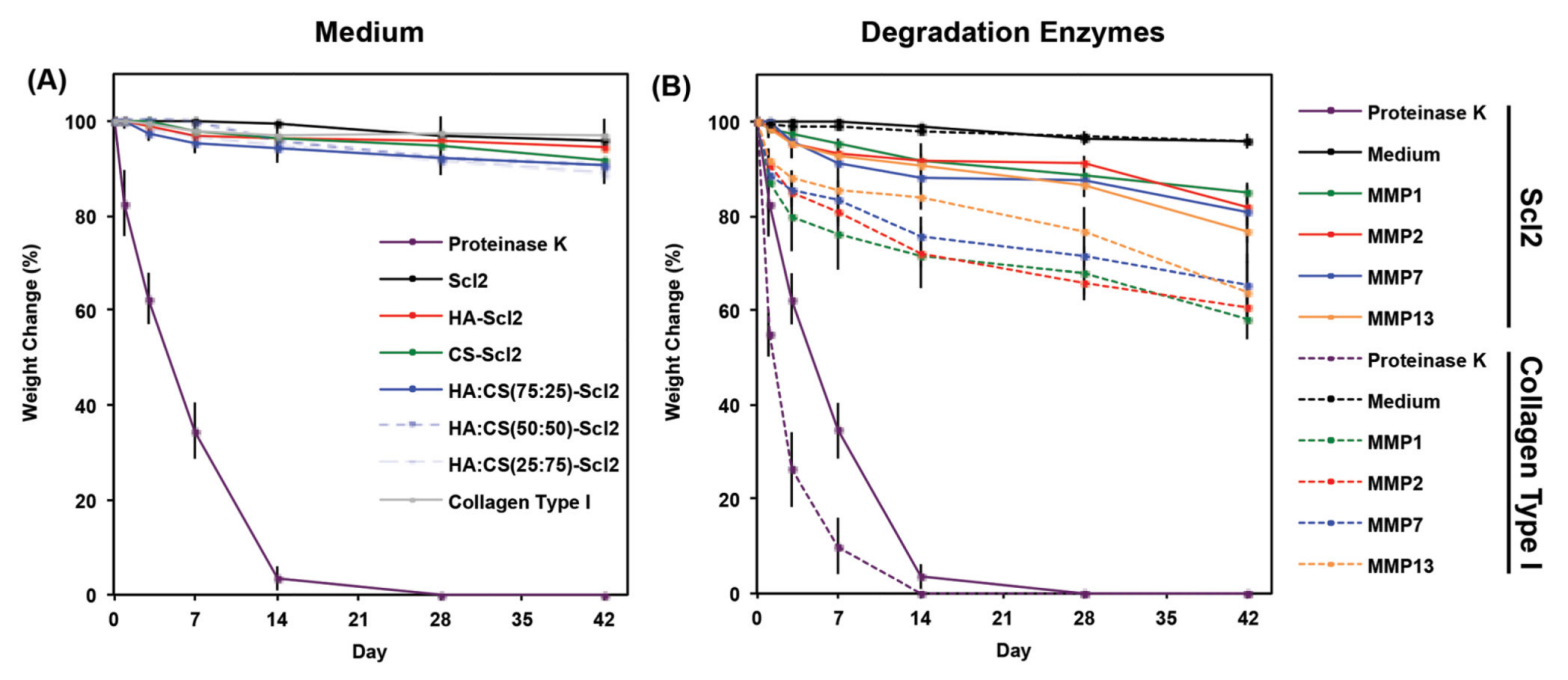
Degradation profile of acellular collagen foams. Degradation of collagen foams incubated in A) chondrogenic medium and B) chondrogenic medium supplemented with recombinant human MMP1, 2, 7, or 13 (30 ng mL^−1^) over time characterized as dry weight loss and expressed as a percentage of initial dry weight. Proteinase K-driven degradation is used as a positive control. Chondrogenic medium without exogenous enzymes is used as a negative control. Values represent means ± SD (*n* = 3).

All foams displayed minimal weight change (less than 10%) over 6 weeks in medium (Figure 3A). The physical crosslinks interlocking the collagen molecules in the foams likely held the structure together. After incubation with enzymes known to be secreted during chondrogenesis of hMSCs (MMP1, MMP2, MMP7, or MMP13)[43,50,56] a significantly increased weight change of the foams was observed compared to medium alone over 6 weeks (Figure 3B). This is likely because MMPs can be promiscuous and are known to recognize and cleave a range of amino acid sequences.[50,56–58] The bacterial collagen foams had a weight reduction of ≈15.2%–23.4% over 6 weeks in the presence of MMPs. Interestingly, the collagen type I foams had a weight reduction of ≈34.6%–41.8% depending on the MMP treatment. It is not surprising that the collagen type I foams degraded faster than those made of bacterial collagen because bacterial collagens are not designed to contain known enzyme-specific cleavage sites while collagen type I has multiple enzymatically targeted sites.[34,41–43] We postulate that these observations may translate positively to the in vivo environment, where a slower degradation rate of the bacterial collagen foams might represent an advantage in allowing increased time for guided matrix accumulation and reorganization compared to collagen type I foams.[43,50,59–61] It is also possible to incorporate specific MMP-cleavable peptide sequences within the Scl2 backbone to tune the degradation of the foams.[43,50,56]

### 2.2 Cellular Response to Collagen Foams

#### 2.2.1 Cell Adhesion and Viability

**Figure 4:**
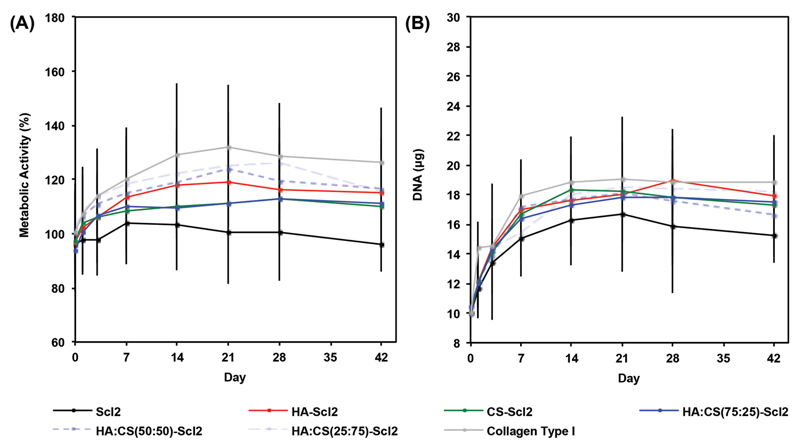
Metabolic activity and DNA content of hMSCs cultured in collagen foams. A) hMSC metabolic activity in collagen foams over 6 weeks in culture. B) DNA content of hMSCs per construct in collagen foams over 6 weeks in culture. All data normalized to day 0. Values represent means ± SD (*n* = 3 for each donor; 3 different bone marrow-derived hMSC donors).

The metabolic activity and DNA content of hMSCs were maintained at high levels in all foams and increased throughout the culture period in all conditions with the exception of the metabolic activity of hMSCs cultured in blank Scl2 foams (Figure 4). The metabolic activity and DNA content of hMSCs were not significantly different between the collagen type I foams and bacterial collagen foams incorporating HA-binding and/or CS-binding sequences at all-time points. Additionally, there was no exogenous supply of GAGs during in vitro culture and thus, metabolic activity of hMSCs was measured in the presence of an endogenous supply of GAGs. The blank Scl2 foams, however, did have a lower metabolic activity and DNA content at day 42 compared to the collagen type I foams. The difference between collagen type I and blank Scl2 foams may be explained by the bioactive sites present in the collagen type I backbone, including integrin-binding sites.[43,44,62–66] Such bioactive sites are not present in the blank Scl2 foams and therefore, additional selected bioactivity has been programmed into the blank Scl2 backbone via the incorporation of the GAG-binding peptides, demonstrating a high degree of control and modularity over the collagen type I foams. The HA-binding and/or CS-binding sequences used in this study have previously been shown to improve cell viability and impact biological processes.[43] The goal of the bacterial collagen is to provide an interface for the retention of GAGs produced by the cells. Our results suggest that this is sufficient to encourage cells to populate the functionalized foams. Cell viability in the different foams was further confirmed by a LIVE/DEAD assay at day 42 (Figure S6, Supporting Information).

#### 2.2.2 In Vitro Chondrogenesis

**Figure 5:**
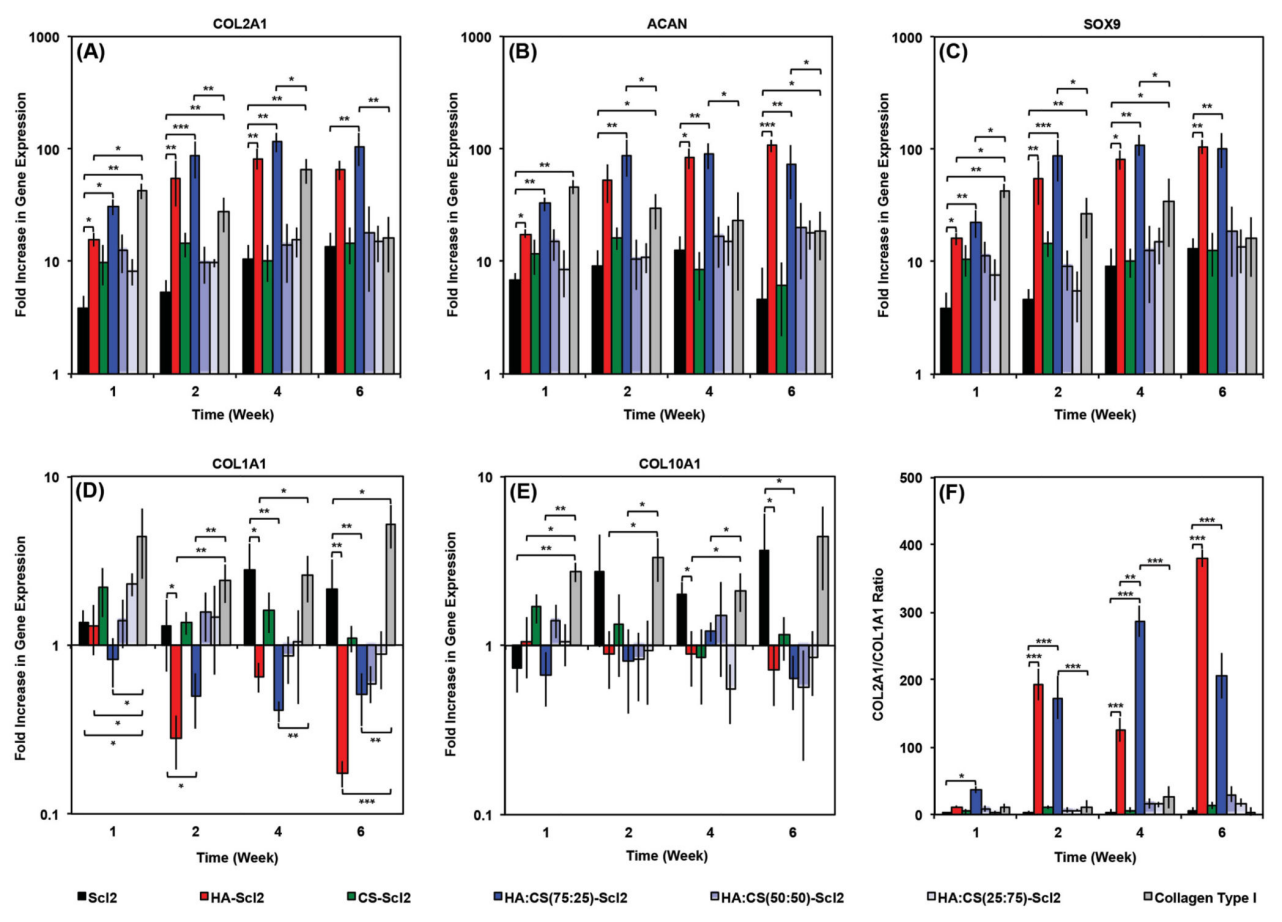
hMSC gene expression in collagen foams. A) COL2A1, B) ACAN, C) SOX9, D) COL1A1, and E) COL10A1 gene expression of hMSCs seeded in collagen foams over 6 weeks in culture, as analyzed using the ΔΔCt method. Data presented as a fold difference relative to undifferentiated hMSCs (calibrator) prior to seeding and normalized to GAPDH (housekeeping gene). F) COL2A1/COL1A1 gene expression ratio. Values represent means ± SD. *p < 0.05, **p < 0.01, ***p < 0.001 (*n* = 3 for each donor; 3 different bone marrow-derived hMSC donors).

The ability to modulate the chondrogenic differentiation of hMSCs seeded within Scl2 foams functionalized with HA-binding and/or CS-binding peptides was evaluated compared to the collagen type I and blank Scl2 foams.[43,44,62] The gene expression of chondrogenic markers (COL2A1, ACAN, and SOX9) (Figure 5A–C) indicated that collagen type I foams significantly enhanced chondrogenic differentiation of hMSCs at an early time point (week 1) compared to the blank Scl2 foams. Furthermore, COL2A1 and SOX9 gene expression by hMSCs in the collagen type I foams were significantly upregulated compared to other functionalized Scl2 foams at week 1. However, the expression profiles of all three chondrogenic marker genes plateaued from week 2 to week 6 for hMSCs seeded in collagen type I foams at which point COL2A1 gene expression of cells in the collagen type I foams was not significantly different to that of cells in the blank Scl2 foams. Interestingly, hMSCs in the HA:CS(75:25)-Scl2 foams had significantly higher COL2A1, ACAN, and SOX9 gene expression at all-time points from week 2 onwards compared to those in the collagen type I foams. Similarly, significantly higher expression of the chondrogenic marker genes was also observed compared to hMSCs in the HA-Scl2 foams at week 2 and week 4. HA-binding and CS-binding peptides incorporated in the bacterial collagen molecules displayed different extents of chondrogenic differentiation of hMSCs despite their similar charge. It has previously been shown that the GAG-binding peptides selected in this study can be used to dynamically and spatially organize the GAG molecules in the same system, suggesting that these peptides interact with endogenous GAG molecules via specific peptide–GAG interactions rather than electrostatic interactions.[43,44] The HA-binding peptide sequence is derived from a HA-binding region of the link protein involved in the stabilization of interactions between HA and aggrecan in native articular cartilage[44] and has been shown to significantly promote the chondrogenesis of hMSCs.[43] The CS-binding peptide sequence is derived through phage display and has previously been shown to noncovalently and specifically bind CS. It is possible that the combination of the HA-binding and CS-binding peptides led to a positive synergistic effect on cell behavior by mimicking native-like protein–GAG interactions. hMSCs in the HA-Scl2 foams exhibited significantly higher COL2A1 and ACAN gene expression at all-time points, and SOX9 at week 2 to week 6 compared to those seeded in the CS-Scl2 foams.

Gene expression levels of COL1A1 and COL10A1 (Figure 5D,E) were significantly higher for hMSCs seeded in blank Scl2 and collagen type I foams compared to all other foams at week 6. Furthermore, COL1A1 and COL10A1 gene expression by hMSCs in these two types of foams were upregulated unlike all other foam formulations, where they remained at basal levels or were downregulated. These results suggest that a controlled functionalization approach through the use of selected modifications to Scl2 delayed or inhibited terminal differentiation of hMSCs. Terminal chondrocyte differentiation of hMSCs toward a hypertrophic state is a critical challenge in cartilage tissue engineering as it can lead to calcification of the newly deposited tissue.[43,67,68] COL2A1/COL1A1 gene expression ratio for the HA-Scl2 and HA:CS(75:25)-Scl2 foams remained high compared to the blank Scl2 and collagen type I foams over 6 weeks of culture (Figure 5F), possibly limiting the fibrochondrocyte phenotype, which is also problematic for the long-term outlook of cartilage repair.[1,3,62,63] Collagen type I has multiple biological moieties including integrin-binding sites that may be interfering with the extent of chondrogenesis and promoting terminal differentiation and fibrochondrocyte phenotype compared to HA-Scl2 and HA:CS(75:25)-Scl2 foams.[43,44,69] Our results indicate that this is a consequence of both an upregulation of collagen type II and a downregulation of collagen type I gene expression by hMSCs in the functionalized foams compared to the blank Scl2 and collagen type I foams. Based on previous findings,[43] we do believe that the GAG-binding peptides contribute to this effect as elaborated in more details in that study. However, we also think that this positive effect is a response to the absence of bioactive motifs in functionalized Scl2 molecules that are not specific to chondrogenesis and are present in collagen type I. This is a major advantage of our system as we have the ability to incorporate only selected bioactive molecules.

**Figure 6:**
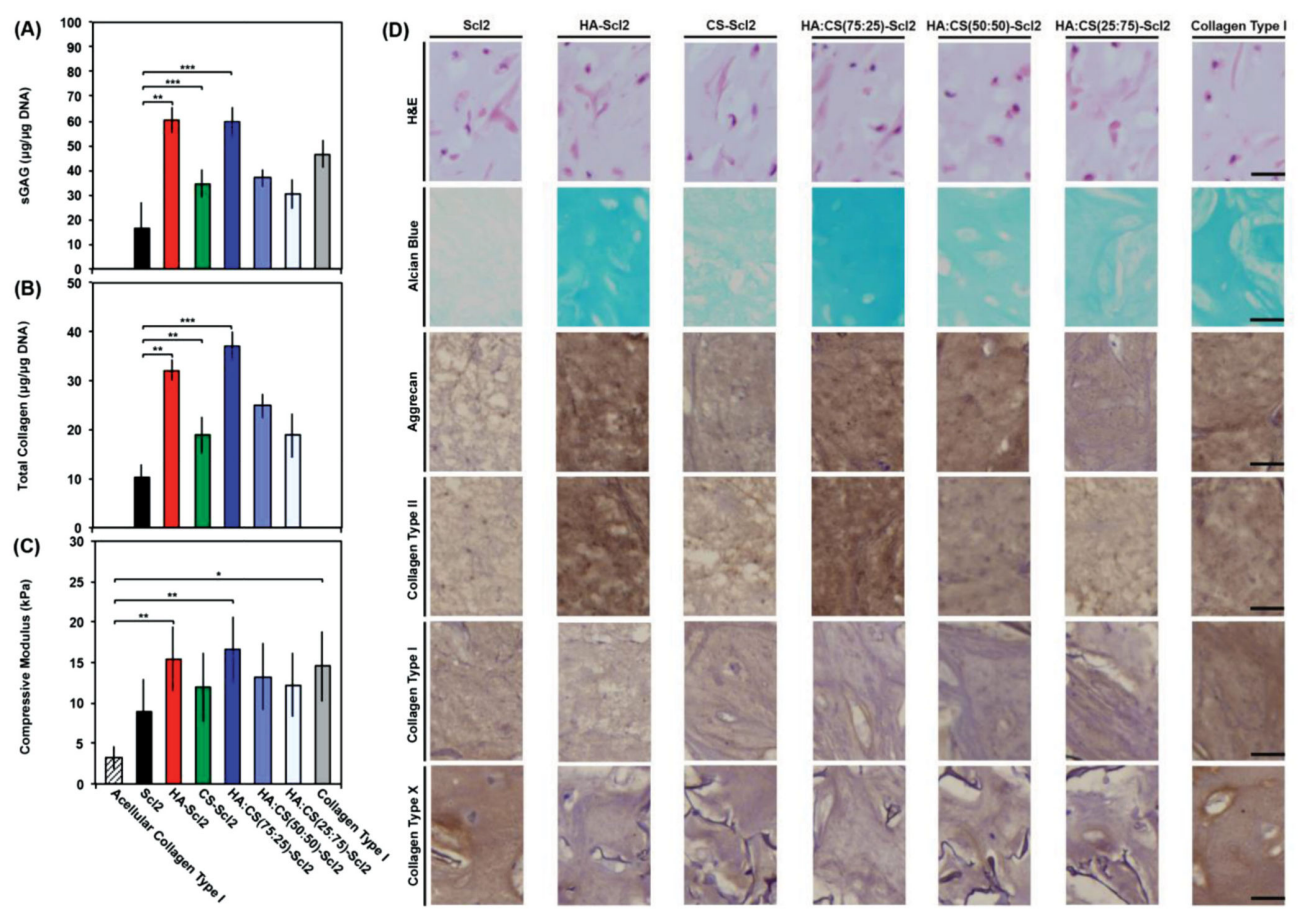
ECM accumulation and compressive modulus of hMSC-seeded collagen foams after 6 weeks of culture. A) Sulfated glycosaminoglycan (sGAG) content of tissue deposited by hMSCs in collagen foams after 6 weeks in culture. B) Hydroxyproline content of tissue deposited by hMSCs in collagen foams after 6 weeks in culture as an estimation of total collagen content. C) Dynamic mechanical analysis (DMA) used to determine the elastic modulus of compression of hMSC-seeded collagen foams compressed to 10% strain at 0.5% strain min^−1^ and 1 Hz after 6 weeks in culture. Values represent means ± SD. *p < 0.05, **p < 0.01, ***p < 0.001 (*n* = 3 for each donor; 3 different bone marrow-derived hMSC donors). D) Representative histological and immunohistochemical examination of hMSC-seeded collagen foams after 6 weeks in culture. Collagen foams are stained with hematoxylin and eosin (H&E), alcian blue for sGAG, and by immunohistochemistry for collagen type I, collagen type II, collagen type X, and aggrecan, from top to bottom. Scale bars are 25 μm.

The hMSC-seeded foams cultured for 6 weeks were further analyzed for ECM accumulation. sGAG content normalized to DNA (Figure 6A) was significantly higher for the HA-Scl2 and HA:CS(75:25)-Scl2 foams compared to the blank Scl2 and collagen type I foams at week 6. Total collagen content normalized to DNA (Figure 6B) was also significantly higher for the HA-Scl2 and HA:CS(75:25)-Scl2 foams compared to the blank Scl2 foams at week 6. Total collagen was not assessed for collagen type I foams because the foams contain hydroxyproline, unlike Scl2, which would have biased the results. These results further validated the benefit of tunable Scl2 proteins as a base material to engineer foams that elicit specific cell-modulating behavior compared to collagen type I foams currently used in the clinic. The increased ECM accumulation also translated to higher compression moduli for the HA-Scl2 and HA:CS(75:25)-Scl2 foams compared to the blank Scl2 and collagen type I foams (Figure 6C and Figure S7, Supporting Information). Similarly, all foam formulations gained weight throughout the culture period, suggesting that ECM accumulation occurred at a faster rate than foam degradation (Figure S8, Supporting Information). Histological evaluation of the foams demonstrated a homogeneous distribution of cells and ECM for all foam types after 6 weeks in culture (Figure 6D). Extensive sGAG accumulation (Alcian Blue staining) was highly noticeable in the HA-Scl2 and HA:CS(75:25)-Scl2 foams. Immunohistochemical analysis of collagen type II and aggrecan confirmed the gene expression results (Figure 6D) for all foams. In fact, collagen type II and aggrecan stainings were markedly greater in the HA-Scl2 and HA:CS(75:25)-Scl2 foams compared to the blank Scl2 and collagen type I foams. Staining for collagen type I and collagen type X (Figure 6D) also confirmed the gene expression profiles for all foam formulations. Specifically, staining for collagen type I and collagen type X in collagen type I foams was much greater compared to the HA-Scl2 and HA:CS(75:25)-Scl2 foams.

#### 2.2.3 Cell–Foam Interactions

**Figure 7:**
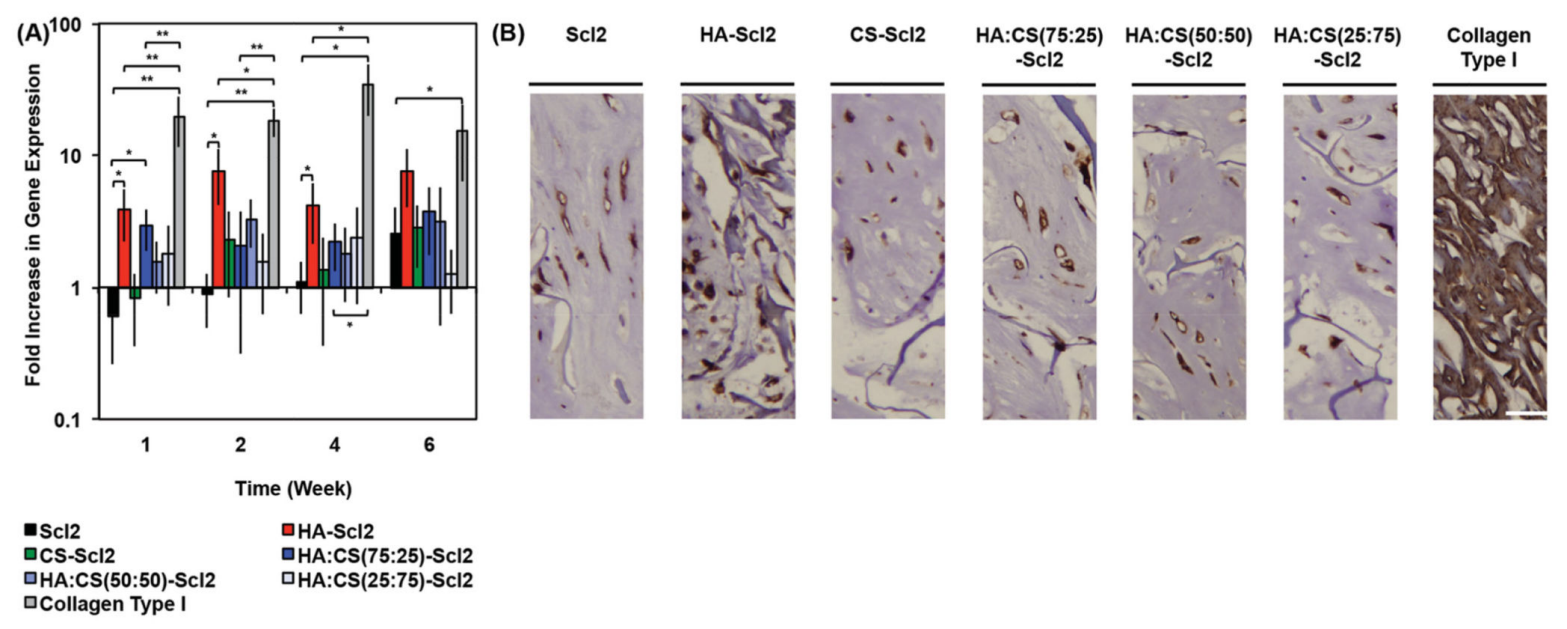
CD44 gene expression in hMSCs cultured in collagen foams. A) CD44 gene expression by hMSCs cultured in collagen foams over 6 weeks, as analyzed using the ΔΔCt method. Data presented as a fold difference relative to undifferentiated hMSCs (calibrator) prior to seeding and normalized to GAPDH (housekeeping gene). Values represent means ± SD. *p < 0.05, **p < 0.01, ***p < 0.001 (*n* = 3 for each donor; 3 different bone marrow-derived hMSC donors). B) Immunohistochemical examination of tissues in collagen foams for CD44 after 6 weeks of culture. Scale bars are 25 μm.

Interestingly, GAG-binding results showed that the HA-Scl2, HA:CS(75:25)-Scl2, and collagen type I foams all exhibited similar levels of HA binding (Figure 2A); however, the extent of chondrogenesis of hMSCs seeded within these bacterial collagen foams was significantly different to the collagen type I foams (Figure 5 and Figure 6). It is unclear whether this effect is due to additional bioactive moieties present on collagen type I molecules or to the biomimetic interactions between HA and the HA-binding peptide selected for this study. To investigate the role of HA binding, we assessed the expression of CD44, a known HA-binding cell surface receptor.[70–72] CD44 expression has been associated with inflammation and has been shown to negatively impact chondrogenesis when expressed at high levels.[73,74] hMSCs in the collagen type I foams had significantly higher CD44 gene expression levels compared to those in the HA-Scl2 and HA:CS(75:25)-Scl2 foams throughout the culture period (Figure 7A). Immunohistochemical analysis of CD44 correlated with the CD44 gene expression results for all foams (Figure 7B). Additional immunohistochemical analysis confirmed the presence of HA in all foams (Figure S9, Supporting Information) and correlated with Figure S6 (Supporting Information). It is not possible to infer the contribution of the specific and biomimetic interaction between HA and the HA-binding peptide on chondrogenesis from these results on CD44 expression. However, these results do suggest that this specific binding interaction may elicit a different cell response than that produced by nonspecific binding with collagen type I. The CD44 gene expression levels present in the HA:CS(75:25)-Scl2 foams were lower compared to the HA-Scl2 foams. This could possibly be due to endogenous CS in the HA:CS(75:25)-Scl2 foams interfering with native HA–CD44 interactions.[70–73] Interestingly, these results also correlate with higher chondrogenic marker gene expression levels by hMSCs in the HA:CS(75:25)-Scl2 foams compared to the HA-Scl2 foams.

## 3 Conclusion

In this work, we have designed and synthesized novel highly porous foams based on recombinant bacterial collagen-mimetic proteins that can be easily and specifically tuned to recreate the complex biochemical microenvironment of native articular cartilage and encourage regeneration. The backbone of blank slate bacterial collagen was modified with varying ratios of specific HA-binding and/or CS-binding sequences and processed to form foams by lyophilization, and dehydrothermal crosslinking. The addition of GAG-binding peptides significantly promoted the chondrogenesis of hMSCs in the bacterial collagen foams compared to blank Scl2 and collagen type I foams. Specifically, hMSCs in the HA-Scl2 and HA:CS(75:25)-Scl2 foams had significantly higher COL2A1, ACAN, and SOX9 gene expression from week 2 onwards resulting in the greatest sGAG and total collagen accumulation and increased compression moduli compared to the collagen type I foams. Taken together, these results demonstrate the high degree of versatility of bacterial collagen foams compared to collagen type I foams and highlight the potential of introducing multiple specific binding sequences within a single system to promote targeted cellular processes. Our novel bacterial collagen system provides a universal platform that can be easily adapted for other regenerative medicine and tissue engineering applications.

## 4 Experimental Section

### Materials

All primary and secondary antibodies used for immunohistochemistry were purchased from Abcam (UK). All other chemicals were purchased from Sigma–Aldrich (UK). All chemicals were used as provided by the manufacturers.

### Streptoccocal Collagen–Like 2 Protein Synthesis and Purification

The gene constructs used were based on the DNA sequence for the fragment of the *Scl2.28* allele (Q8RLX7) of *Streptococcus pyogenes* encoding the combined globular and collagen-like portions of the *Scl2.28* protein, but lacking the N and C termini attachment domains as previously described.[40–42,45] Constructs included an additional enzyme cleavage and spacer sequence LVPRGSP between the N terminal globular domain (V) and the following (Gly–Xaa–Yaa)_n_ collagen-like (CL) domain sequences. The Scl2 (VCL) construct used as a control was as previously described.[40–42,45] The other two constructs contained HA-binding (RYPISRPRKR) or CS-binding (YKTNFRRYYRF) peptides integrated in the structure. These constructs (HA-Scl2 and CS-Scl2) comprised two CL domains with the peptide sequences inserted between the domains (Figure 1). The C terminal of these constructs had an additional GGPCPPC sequence. Subsequently, in order to further stabilize the triple helix an additional GGPCPPC sequence was also added at the N terminal between the spacer sequence and the CL domain, using the QuikChange site-directed mutagenesis kit (Stratagene) according to the manufacturers’ instructions and synthesised primers (Integrated DNA Technologies). All DNA sequences were synthesized commercially with codon optimization for expression in *E. coli* (GeneArt Gene Synthesis, Germany). The sequences of all constructs were confirmed by sequencing prior to transformation and protein expression.

DNA sequences were subcloned into the pColdI (Takara Bio, Japan) vector systems for expression in *E.coli*. The constructs did not include an N terminal His_6_-tag, which was provided by the pColdI vector.[40–42] For protein production, a selected positive clone was transformed and then expanded in flask culture. The pColdIII constructs were expressed in *E. coli* BL21-DE3 strain. Cells were grown in 2 x yeast extract-tryptone (YT) media with ampicillin (100 μg mL^−1^) at 37 °C, shaking at 200 rpm until the A600 absorbance reading reached an optical density in the range 3–6 A.U. Cells were then cooled to 25 °C and isopropyl *β*-d-thiogalactopyranoside (1 × 10^−3^ m) was added to induce protein expression. After 10 h incubation, cells were further cooled to 15 °C for 14 h, after which the cells were harvested by centrifugation (12 000 g, 60 min) at 4 °C. For protein extraction, cell paste (1 g) was resuspended in sodium phosphate buffer (20 mL, 20 × 10^−3^ m) at pH 8.0 and the cells ruptured using either a French press or by sonication on ice, using a Misonix S4000 instrument, with a Enhance Booster #1 probe.[40–42,45] Clarified lysate (12 000 g for 30 min, 4 °C) was adjusted to pH 2.2 and held at 4 °C for 16 h. Any precipitate that had formed was removed (12 000 g for 30 min, 4 °C) and the supernatant, containing the expressed collagens, was treated by pepsin (0.01 mg mL^−1^) for 16 h at 4 °C.[45] Collagens were concentrated and buffer exchanged into sodium phosphate buffer (20 × 10^−3^ m), pH 8.0 using a 10 kDa cross-flow filtration membrane (Pall Life Sciences). Purity was verified by 12% sodium dodecyl sulfate–polyacrylamide gel electrophoresis (SDS–PAGE)[40–42] and matrix assisted laser desorption spectroscopy (MALDI; Waters).

### Preparation of Foams

To generate foams, all protein samples were dissolved in acetic acid (10 mg mL^−1^, 50 × 10^−3^ m) at room temperature, and the pH was adjusted to 7.4 using NaOH (1 m). To generate HA:CS(75:25)-Scl2, HA:CS(50:50)-Scl2, and HA:CS(25:75)-Scl2 foams, HA-Scl2 and CS-Scl2 proteins were mixed in a 75:25, 50:50, and 25:75 molar ratio, respectively. Blank Scl2 and rat tail collagen type I proteins were used as controls. The solutions were sterile-filtered and pipetted into 8 mm diameter by 2 mm thickness custom-made polydimethylsiloxane molds. The samples were frozen at −20 °C for 24 h, transferred to −80 °C for 4 h, and lyophilized for 8 h. The samples were then subjected to a dehydrothermal cross-linking treatment where they were heated to 121 °C for 24 h under high vacuum conditions to minimize protein denaturation.[46–48] During the dehydrothermal crosslinking treatment, water was removed between the collagen molecules resulting in the formation of physical intermolecular cross-links through condensation reactions via amide formation or esterification. Foams were sterilized by washing three times with 70% (v/v) ethanol for 20 min each followed by three washes with phosphate buffered saline (PBS) for 20 min each.

### Morphological Characterization

Cross-sectional morphological examination of all foams in a dry state was performed. Foams were imaged by scanning electron microscopy (SEM) using a JEOL 5610 (Herts, UK). Samples were coated with 100 Å Au using an Emitech K550 sputter coater prior to imaging at an accelerating voltage of 15 kV and a working distance of 150 mm.

Foams were incubated in PBS, 0.5 mg mL^−1^ HA (Creative PEGWorks, UK), or 0.5 mg mL^−1^ CS (Creative PEGWorks, UK) for 24 h at 37 °C in a 5% CO_2_ atmosphere, washed three times in PBS and imaged by multiphoton second harmonic generation (MP–SHG) in wet state using a Leica SP5 inverted microscope equipped with a MaiTai HP DeepSee multiphoton laser (Spectraphysics) on a 25× NA objective. Second harmonic signal was generated at 900 nm and detected on a photomultiplier tube (PMT) (435–465 nm). Collagen samples were further characterized using Fourier transform infrared (FTIR) spectroscopy. FTIR analysis with a PerkinElmer Spectrum One spectrometer was used to determine FTIR spectra showing characteristic peaks for all samples. FTIR spectra were taken with a scanning wavenumber range from 4000 to 650 cm^−1^.

### Mechanical Characterization

Mechanical properties of the foams were studied using dynamic mechanical analysis (DMA) in unconfined compression mode. For all tests, samples were incubated in PBS for 30 min prior to testing and dimensions were measured in wet state using digital calipers. Foams were mechanically tested in compression using a Bose Electroforce testing machine equipped with a 22.5 N load cell. Samples were incubated in PBS, preloaded to 0.05 N, compressed to 10% strain at a crosshead speed of 0.5% strain min^−1^, and then followed by a frequency sweep from 0.1 to 10 Hz. The compressive modulus was calculated from the linear portion of the stress–strain curve.

### GAG Binding

The effectiveness at binding and retaining specific GAGs on the HA-binding and the CS-binding peptide sequences that had been incorporated in the Scl2 backbone was evaluated using fluorescein isothiocyanate (FITC)-labeled HA and CS (Creative PEGWorks, UK). Scl2 and collagen type I proteins were coated onto 96-well plates, incubated at 37 °C for 24 h, washed three times in PBS, incubated in bovine serum albumin (BSA) (1% (w/v)) in PBS for 5 h, washed three times in PBS, incubated in FITC-labeled HA or CS (0.5 mg mL^−1^) in PBS for 24 h, washed three times in PBS to remove unbound fluorescent GAGs, and stored in PBS at 37 °C between measurements. To study the release of HA or CS from the foams, foams were processed exactly as protein-coated wells. After 1, 3, and 7 d, PBS was removed and fluorescence intensities of the supernatant were measured to evaluate GAG binding and retention. Samples were excited at 485 or 535 nm, and the fluorescence emission intensity was measured at 525 or 620 nm for FITC-labeled HA or CS, respectively. Samples were incubated in fresh PBS at 37 °C between readings. The fluorescence was measured in arbitrary units and relative binding of HA or CS was normalized to the highest level of fluorescent intensity at each time point. PBS and FITC-labeled HA or CS (0.5 mg mL^−1^) in PBS were used as negative and positive controls, respectively.

### Swelling Behavior

The swelling behavior of foams was measured by recording their dry weight (*W*_d_) followed by swelling in HA or CS (0.5 mg mL^−1^) in PBS for 24 h at 37 °C. The foams were then removed from solution at 2, 4, 6, 24, and 48 h post incubation, washed three times in PBS, and their wet weight (*W*_s_) was recorded. The swelling ratio of the foams was calculated using Equation (1) and normalized to their swelling ratio in PBS without exogenous GAGs in the solution.

(1) $$\text{Swelling}\;\text{ratio}(\%)=\left[\frac{\left(w_{s}-w_{d}\right)}{w_{d}}\right]\times 100$$

### hMSC Culture

Bone marrow-derived hMSCs were purchased from PromoCell GmbH (Germany). hMSCs were seeded at 4000 cells per cm^2^ in T225 flasks and cultured in mesenchymal stem cell growth medium (MSCGM) (PromoCell GmbH, Germany). hMSCs were incubated at 37 °C in a 5% CO_2_ atmosphere and the medium was changed every 3 d. The cells were harvested at 80% confluency with trypsin–EDTA (0.025% (w/v)) in PBS, centrifuged, and subcultured in MSCGM. Passage 6 hMSCs were used for all cell–material interaction experiments.

### Cell Seeding and Culture

Foams were placed in 48-well plates coated with agarose (2% (w/v)) to prevent cells from adhering to the well surfaces. An aliquot (50 μL) of chondrogenic medium (high-glucose (4.5 g L^−1^) Dulbecco’s modified Eagle medium (DMEM; Invitrogen, UK) supplemented with dexamethasone (0.1 × 10^−3^ m), penicillin streptomycin (1% (v/v)), l-proline (50 μg mL^−1^), ascorbate-2-phosphate (50 μg mL^−1^), 1× insulin–transferrin–selenium (ITS) Premix (BD Biosciences, UK), and TGF-*β*3 (Lonza, UK) (10 ng mL^−1^)) containing 1 × 10^6^ hMSCs was injected into the foams and the cells were allowed to migrate and adhere to the foams for 30 min at 37 °C in a 5% CO_2_ atmosphere before slowly topping up the wells with chondrogenic medium. Foams were incubated for 6 weeks with the medium changed every 3 d.

### Weight Change

The dry weight of each foam was measured over time in the absence of cells to evaluate their degradation. Foams were prepared as previously described and incubated in chondrogenic medium for 24 h at 37 °C in a 5% CO_2_ atmosphere. The foams were incubated in chondrogenic medium with exogenous MMPs (30 ng mL^−1^) at 37 °C in a 5% CO_2_ atmosphere for 1 week with medium changed and dry weight measurements taken daily. Percentage weight change was normalized to day 0. Degradation by recombinant human MMP1, MMP2, MMP7, and MMP13 (AnaSpec, USA) was tested against a negative control (chondrogenic medium alone) and a positive control (0.2 μg mL^−1^ proteinase K).

Cell-seeded foams were incubated in chondrogenic medium at 37 °C in a 5% CO_2_ atmosphere for 6 weeks, and dry weight measurements were taken after 0, 1, 3, 7, 14, 21, 28, and 42 d of culture. Percentage weight change corresponding to the cumulative effect of cell proliferation, cartilage-like matrix deposition, and foam degradation was normalized to day 0.

### Cell Adhesion and Viability

hMSC-seeded foams were cultured for 0, 1, 3, 7, 14, 21, 28, and 42 d. After the culture period, the foams were washed three times in PBS and analyzed for cell adhesion and viability. Cell viability was qualitatively assessed with a LIVE/DEAD Viability/Cytotoxicity Kit (Molecular Probes, USA) according to the manufacturers’ instructions. Fluorescence confocal microscopy (Leica SP5 inverted microscope, Leica Microsystems) was used to visualize live (calcein; green) and dead (ethidium homodimer–1; red) cells. The metabolic activity of cells in the foams was quantified by the AlamarBlue assay (Serotec, USA). This assay is based on the fluorescent signal output produced by metabolically active cells. Measurements were made at 570 nm and 600 nm. Cell-free foams and empty wells were used as controls. All data were normalized to DNA content present at each time point.

### DNA, sGAG, and Hydroxyproline Quantification

hMSC-seeded foams were cultured for 0, 1, 3, 7, 14, 21, 28, and 42 d. After the culture period, the constructs were washed three times in PBS and digested individually in papain digest solution (2.5 units papain per mL, cysteine HCl (5 × 10^−3^ m), EDTA (5 × 10^−3^ m), pH 7.4, in PBS) at 60 °C for 24 h. Papain digests were stored at −20 °C until further analysis. Digested samples were assayed for DNA content using the Quant–iT PicoGreen Kit (Invitrogen, USA) according to the manufacturers’ instructions. Measurements were made at 535 nm. The standard curve was generated with dsDNA (Invitrogen, USA).

Sulfated glycosaminoglycan (sGAG) content was quantified using the Blyscan Kit (Biocolor, UK) according to the manufacturers’ instructions. Measurements were made at 656 nm. The standard curve was generated with bovine trachea chondroitin sulfate A.

Total new, cell-derived collagen content was estimated by measuring the hydroxyproline content. Unlike mammalian collagens, bacterial collagens lack hydroxyproline, which enabled us to distinguish between the collagen in the foams and new collagen deposition by the hMSCs. Papain-digested samples were hydrolyzed in HCl (6 N) at 110 °C for 18 h, then pH adjusted using NaOH (5.7 N). The hydroxyproline content of the hydrolysate was determined using the chloramine-T/Ehrlich’s reagent assay and the color change quantified spectrophotometrically at 560 nm.[49,50] The standard curve was generated with L-hydroxyproline and a conversion factor of 10 was used to convert from hydroxyproline to total collagen content.

### Histology and Immunohistochemistry

After 0 and 42 d of culture, hMSC-seeded foams were washed three times in PBS, fixed with paraformaldehyde (Electron Microscopy Sciences, USA) (4% (v/v)) for 30 min at 4 °C, washed three times in PBS, permeabilized with Triton X-100 (0.4% (v/v)) for 30 min, and washed again. Foams were flash frozen in OCT (Tissue-Tek, Fisher Scientific) and cross-sections were cryosectioned at a thickness of 10 μm. Sections were transferred to treated slides (Superfrost Plus, Thermo Scientific) and allowed to adhere for 24 h at 4 °C. Slides were stained for deposited sGAG with alcian blue (AB; pH 2.5) and for cell nuclei and matrix with hematoxylin and eosin (H&E).

Immunohistochemical staining (IHC) was performed for collagen type I, collagen type II, collagen type X, aggrecan, and CD44 with rabbit IgG secondary antibody and PBS negative controls. Samples were pretreated with hydrogen peroxide (3%), an avidin and biotin blocking kit (Vector Labs, UK), and blocked with goat serum (5% (v/v)). Primary antibodies were incubated overnight at 1/200 in goat serum (5% (v/v)), followed by goat anti-rabbit secondary antibody labeled with HRP at 1:100 for 1 h, stained with a 3,3′-diaminobenzidine (DAB) kit (Vector Labs, UK) for 10 min, and counter-stained with hematoxylin. All stained sections were dehydrated, mounted with Histomount (Fisher Scientific, UK), and viewed on an Olympus BX51 microscope equipped with an Olympus DP70 camera.

### Gene Expression Analysis

hMSC-seeded foams were cultured for 0, 1, 3, 7, 14, 21, 28, and 42 d. After the culture period, the constructs were washed three times in PBS. Total RNA was isolated using a tissue ruptor (Qiagen, USA) to homogenize samples with RLT buffer after which QIAshredder columns (Qiagen, USA) and the RNeasy Mini Kit (Qiagen, USA) were used to extract the RNA according to the manufacturers’ instructions. QuantiTect Reverse Transcription Kit (Qiagen, USA) and QuantiTect SYBR Green PCR Kit (Qiagen, USA) were used to perform reverse transcription and quantitative PCR (qPCR), respectively. Thermocycling and SYBR Green detection were performed on a Corbett Rotorgene 6000 (Qiagen, USA) with extension at 72 °C and denaturing at 95 °C. Annealing temperatures were primer specific. Data were analyzed using the ΔΔ*C*t method.[51] The following primers were used: GAPDH (Quiagen, USA) (Forward 5′-TGGTATCGTGGAAGGACTCATGA-3′ and Reverse 5′-ATGCCAGTGAGCTTCCCGTTCAG-3′), COL1A1 (Forward 5′-CATTAGGGGTCACAATGGTC-3′ and Reverse 5′-TGGAGTTCCATTTTCACCAG-3′), COL2A1 (Forward 5′-CATCCCACCCTCTCACAGTT-3′ and Reverse 5′-GTCTCTGCCTTGACCCAAAG-3′), COL10A1 (Forward 5′-AATGCCTGTGTCTGCTTTTAC-3′ and Reverse 5′-ACAAGTAAAGATTCCAGTCCT-3′), CD44 (Forward 5′-CATCTACCCCAGCAACCCTA-3′ and Reverse 5′-CTGTCTGTGCTGTGGGTGAT-3′), and ACAN (Forward 5′-CACTGTTACCGCCACTTCCC-3′ and Reverse 5′-GACATCGTTCCACTCGCCCT-3′) at an annealing temperature of 60 °C, and SOX9 (Forward 5′-AACGCCGAGCTCAGCAAG-3′ and Reverse 5′-ACGAACGGCCGCTTCTC-3′) at an annealing temperature of 62 °C.

### Statistical Analysis

All cell-related experiments were repeated three times with hMSCs from different donors and with each donor having an intraexperimental sample size of 3. Data are presented as means ± standard deviation (SD). Statistical significance was determined by performing analysis of variance (ANOVA) with Bonferroni correction and with a significance accepted at *p* value < 0.05.

## Supporting Information

Supporting Information is available from the Wiley Online Library or from the author.

Supporting Information
