# Supplementary material for: Harnessing the Versatility of Bacterial Collagen to Improve the Chondrogenic Potential of Porous Collagen Scaffolds
Source: Adv Healthc Mater. Author manuscript; Available in PMC 2017 Apr 26. (PMC5405340; doi:10.1002/adhm.201600136)
Supplement: Supporting Information [file NIHMS72406-supplement-Supporting_Information.pdf]

Copyright WILEY-VCH Verlag GmbH & Co. KGaA, 69469 Weinheim, Germany, 2016.

ADVANCED
HEALTHCARE
MATERIALS

Supporting Information

for *Adv. Healthcare Mater.*, DOI: 10.1002/adhm.201600136

Harnessing the Versatility of Bacterial Collagen to Improve the Chondrogenic Potential of Porous Collagen Scaffolds

*Paresh A. Parmar, Jean-Philippe St-Pierre, Lesley W. Chow, Jennifer L. Puetzer, Violet Stoichevska, Yong Y. Peng, Jerome A. Werkmeister, John A. M. Ramshaw, and Molly M. Stevens**

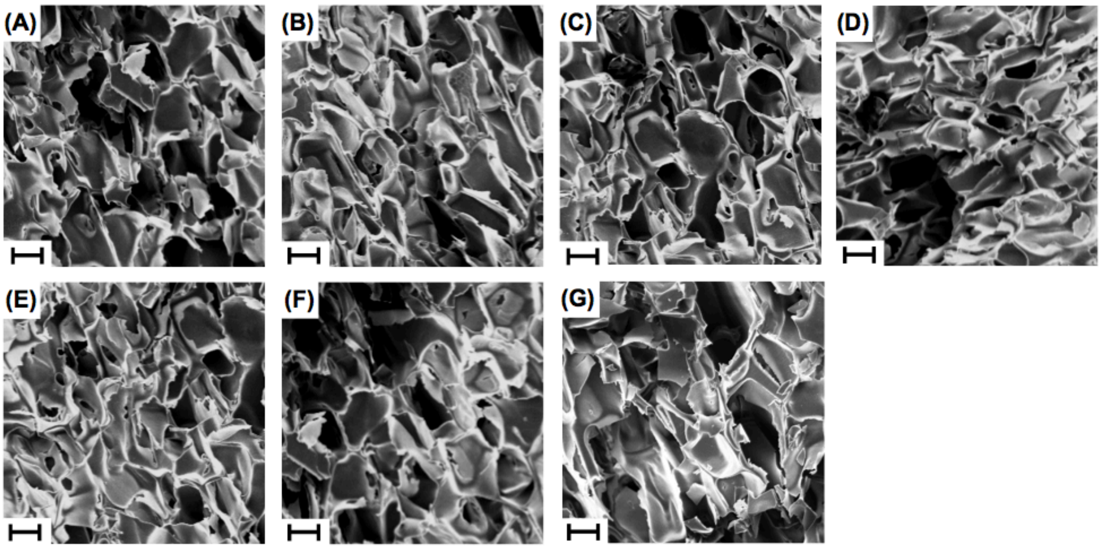

Fig. S1. Representative scanning electron microscopy (SEM) images of acellular (A) Scl2, (B) HA-Scl2, (C) CS-Scl2, (D) HA:CS(75:25)-Scl2, (E) HA:CS(50:50)-Scl2, (F) HA:CS(25:75)-Scl2, and (G) collagen type I foams (scale bars are 100 µm). Frames A and G are reproduced from Fig. 1.

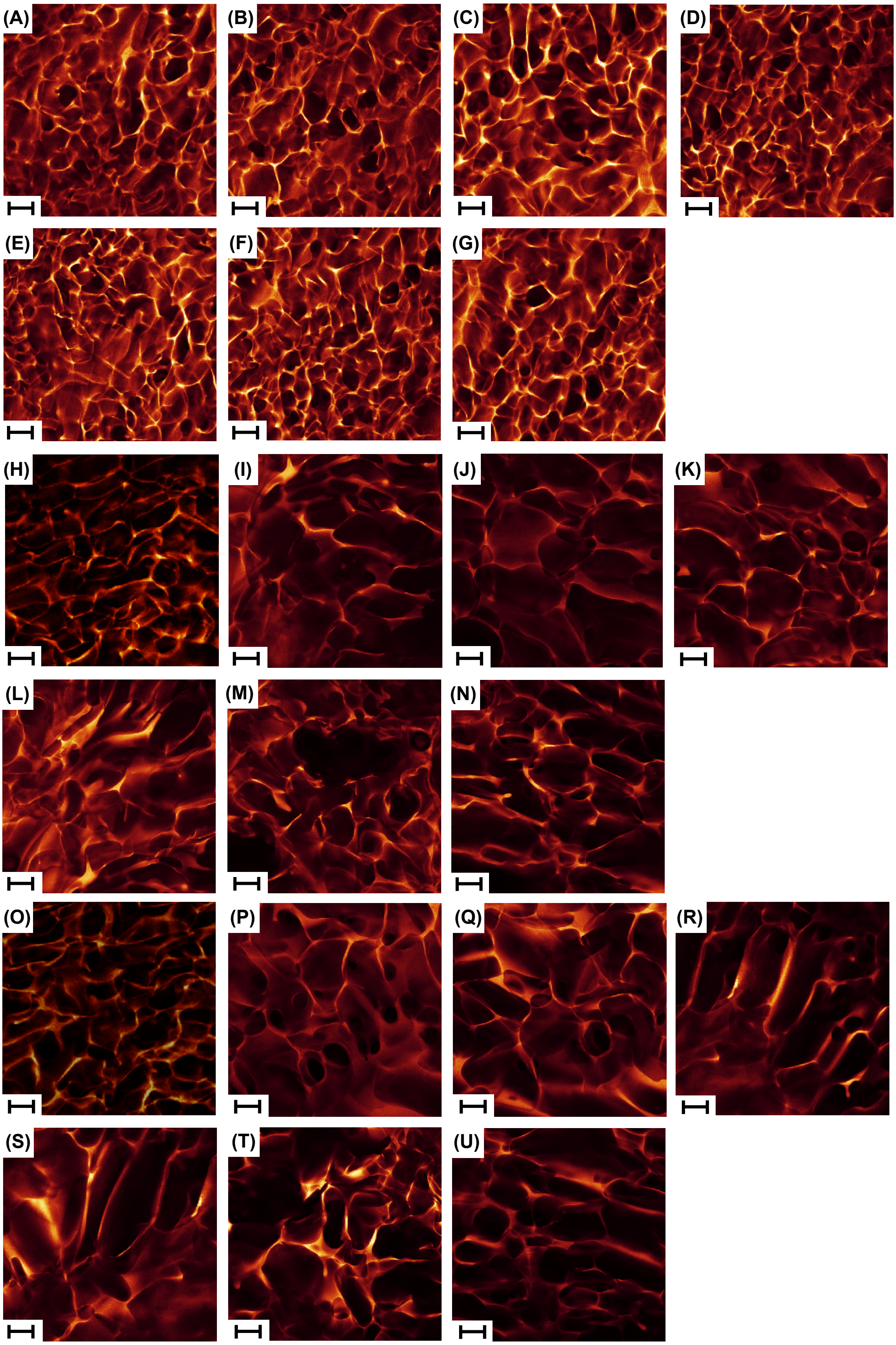

Fig. S2. Representative multi–photon second harmonic generation (MP–SHG) images of acellular foams in the absence of GAGs (A–G), in the presence of HA (H–N), and in the presence of CS (O–U). (A, H, O) Scl2, (B, I, P) HA-Scl2, (C, J, Q) CS-Scl2, (D, K, R) HA:CS(75:25)-Scl2, (E, L, S) HA:CS(50:50)-Scl2, (F, M, T) HA:CS(25:75)-Scl2, and (G, N, U) collagen type I foams (scale bars are 200 μm). Frames A and G are reproduced from **Figure 1**.

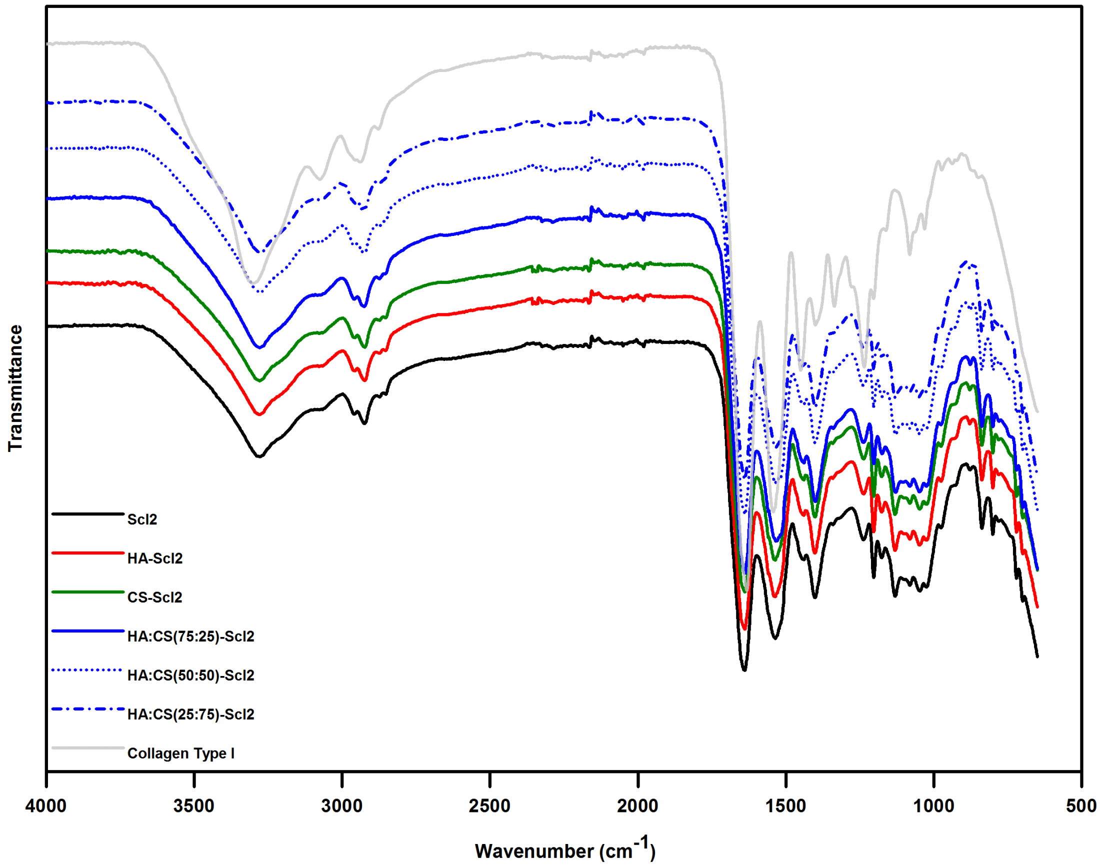

Fig. S3. Representative FTIR spectra of collagen foams.

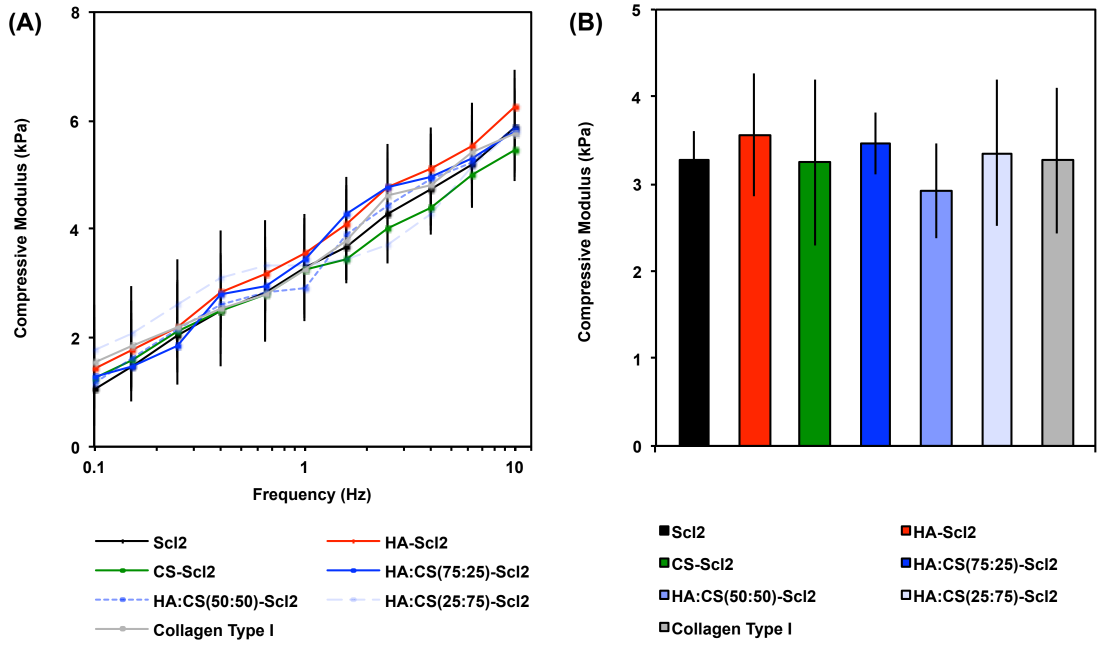

Fig. S4. Dynamic mechanical analysis (DMA). Elastic modulus in unconfined compression of acellular collagen foams compressed to 10% strain at 0.5% strain/min (A) from 0.1 to 10 Hz and (B) at 1 Hz, respectively. Values represent means ± SD (n = 3).

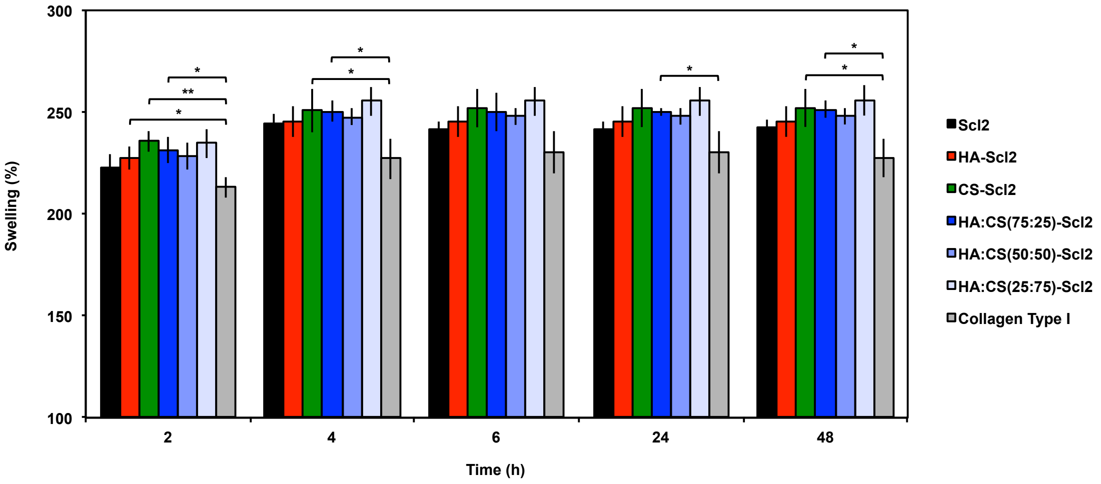

Fig. S5. Swelling behavior of acellular collagen foams without exogenous addition of HA and CS. Values represent means ± SD. $^*p < 0.05$, $^{**}p < 0.01$ (n = 3).

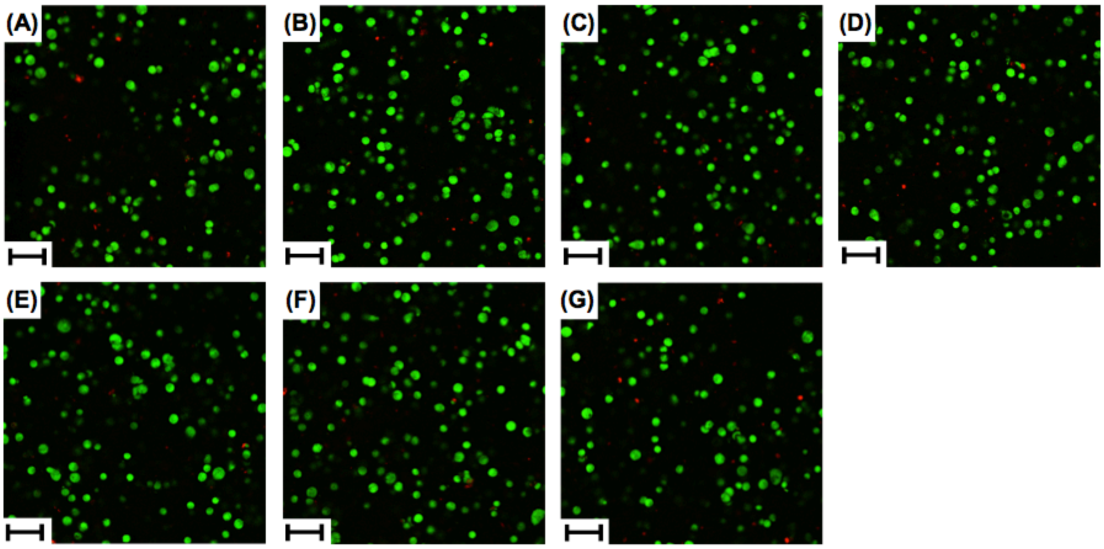

Fig. S6. hMSC viability in collagen foams. LIVE/DEAD® Viability/Cytotoxicity assay on hMSCs cultured for 6 weeks in collagen foams. Representative confocal images of cells taken at the cross–section (depth of 500 μm) in (A) Scl2, (B) HA-Scl2, (C) CS-Scl2, (D) HA:CS(75:25)-Scl2, (E) HA:CS(50:50)-Scl2, (F) HA:CS(25:75)-Scl2, and (G) collagen type I foams (scale bars are 50 μm).

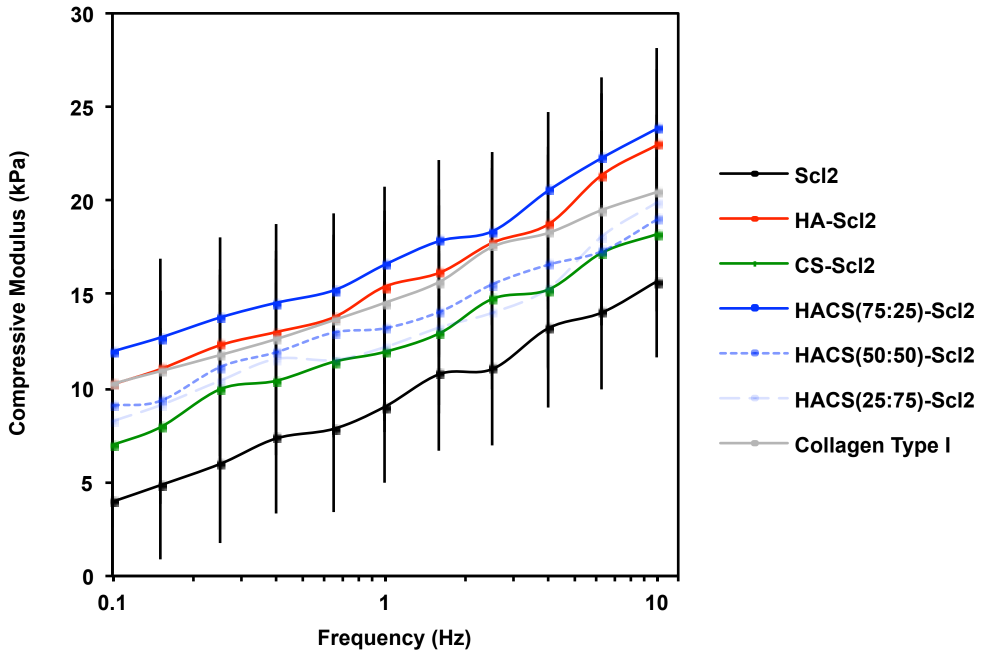

Fig. S7. Dynamic mechanical analysis (DMA). Elastic compression moduli of cell–seeded collagen foams compressed to 10% strain at 0.5% strain/min from 0.1 to 10 Hz after 6 weeks of culture. Values represent means ± SD (n = 3 for each donor; 3 different bone marrow–derived hMSC donors).

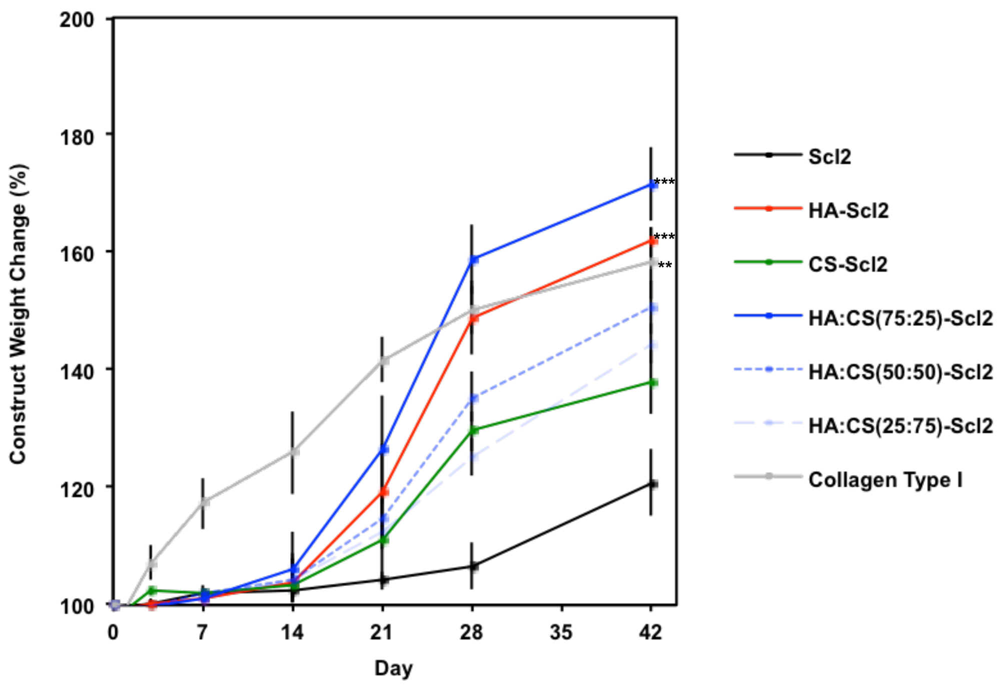

Fig. S8. Collagen foam dry weight change over time in culture with hMSCs. Weight change was normalized to dry weight at day 0. Values represent means ± SD. ***p* < 0.01, ****p* < 0.001 versus Scl2 (n = 3 for each donor; 3 different bone marrow–derived hMSC donors).

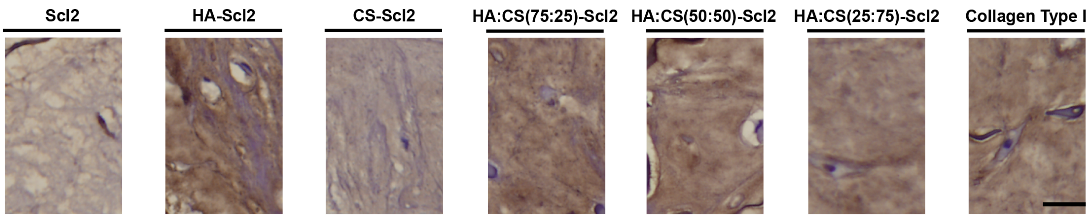

Figure S9. Immunohistochemical examination of tissues in collagen foams for HA after 6 weeks of culture. Scale bars are 25 μm.
